# 1095. Pharmacokinetics-Pharmacodynamics Evaluation of Tebipenem Pivoxil Hydrobromide Using the 10-Day Hollow-Fiber *In Vitro* Infection Model

**DOI:** 10.1093/ofid/ofab466.1289

**Published:** 2021-12-04

**Authors:** Brian D VanScoy, Haley Conde, Sean Jones, Ian A Critchley, Thomas R Parr, Nicole Cotroneo, Paul G Ambrose

**Affiliations:** 1 Institute for Clinical Pharmacodynamics, Inc., Schenectady, New York; 2 Institute for Clinical Pharmacodynamics, Schenectady, New York; 3 Spero Therapeutics, Cambridge, Massachusetts

## Abstract

**Background:**

Tebipenem pivoxil hydrobromide, an orally bioavailable prodrug of tebipenem, is currently in development for the treatment of complicated urinary tract infections. The spectrum of tebipenem activity against Enterobacterales is consistent with intravenous carbapenems such as ertapenem and meropenem including extended spectrum beta-lactamase (ESBL) and AmpC producing organisms. The goal of these studies was to determine the tebipenem exposure required to prevent on-therapy resistance and to evaluate the tebipenem regimen utilized in the recent Phase 3 clinical trial.

**Methods:**

Three *Escherichia coli* clinical isolates (ESBL +, Sequence Type -131) (tebipenem MIC = 0.008 to 0.03 mg/L) were subjected to concentration-time profiles simulating free-drug plasma concentrations after oral administration of tebipenem regimens in a hollow-fiber *in vitro* infection model. Dose-ranging studies were completed using two isolates at an initial burden of 10^8^ CFU/mL and with exposures for tebipenem regimens ranging from 4.69 to 1200 mg administered every eight hours (q8h). An additional isolate was subjected to only the tebipenem 600 mg q8h regimen evaluated in the Phase 3 trial. Samples were collected for the enumeration of bacterial burdens and evaluation of the simulated pharmacokinetic profile. Each sample for bacterial enumeration was suspended onto drug-free and tebipenem-supplemented agar plates in order to observe the density of the total- and drug-resistant subpopulations over the 10-day period.

**Results:**

A full dose response, ranging from treatment failure to reductions in bacterial burden from baseline, were observed in the dose-ranging studies for the two *E. coli* isolates. Tebipenem consistently lowered bacterial burdens below that of the initial inoculum at a dose of 600 mg q8h for all three *E. coli* isolates. Amplification of resistance was observed intermittently for all regimens, but was never equal to that of the total population at the 600 mg q8h clinical dose. Tebipenem MIC values for isolates collected from the drug-supplemented agar ranged from 0.06 to 0.25 mg/L, representing four two-fold dilutions from baseline.

**Conclusion:**

These data support the selection of tebipenem dosing regimens that minimize the potential for on-therapy drug-resistance amplification.

**Disclosures:**

**Brian D. VanScoy, B.S.**, **3-V Biosciences** (Grant/Research Support)**Achogen** (Grant/Research Support)**Amplyx Pharmaceuticals, Inc.** (Grant/Research Support)**Arixa Pharmaceuticals** (Grant/Research Support)**Arsanis Inc.** (Grant/Research Support)**B. Braun Medical Inc.** (Grant/Research Support)**Basilea Pharmaceutica** (Grant/Research Support)**BLC USA** (Grant/Research Support)**Boston Pharmaceuticals** (Grant/Research Support)**Bravos Biosciences, LLC** (Grant/Research Support)**Cidara Therapeutics Inc.** (Grant/Research Support)**Cipla, USA** (Grant/Research Support)**Corcept Therapeutics** (Grant/Research Support)**Cumberland Pharmaceuticals** (Grant/Research Support)**Debiopharm International SA** (Grant/Research Support)**Discuva Limited** (Grant/Research Support)**Emerald Lake Technologies** (Grant/Research Support)**Enhanced Pharmacodynamics** (Grant/Research Support)**Entasis Therapeutics** (Grant/Research Support)**E-Scape Bio** (Grant/Research Support)**Genentech** (Grant/Research Support)**Geom Therapeutics, Inc.** (Grant/Research Support)**GlaxoSmithKline** (Grant/Research Support)**Hoffmann-La Roche** (Grant/Research Support)**Horizon Orphan LLC** (Grant/Research Support)**ICPD Biosciences, LLC** (Grant/Research Support)**Indalo Therapeutics** (Grant/Research Support)**Insmed Inc.** (Grant/Research Support)**Institute for Clinical Pharmacodynamics** (Employee)**Iterum** (Grant/Research Support)**KBP Biosciences USA** (Grant/Research Support)**Kyoto Biopharma, Inc.** (Grant/Research Support)**Matinas** (Grant/Research Support)**Meiji Seika Pharma Co., Ltd.** (Grant/Research Support)**Melinta Therapeutics** (Grant/Research Support)**Menarini Ricerche S.p.A.** (Grant/Research Support)**Merck & Co., Inc** (Grant/Research Support)**Mutabilis** (Grant/Research Support)**Nabriva Therapeutics AG** (Grant/Research Support)**Naeja-RGM Pharmaceuticals** (Grant/Research Support)**Nosopharm SAS** (Grant/Research Support)**Novartis Pharmaceuticals Corp.** (Grant/Research Support)**NuCana Biomed** (Grant/Research Support)**Paratek Pharmaceuticals, Inc.** (Grant/Research Support)**Polyphor, Ltd.** (Grant/Research Support)**Prothena Corporation** (Grant/Research Support)**PTC Therapeutics** (Grant/Research Support)**Rempex Pharmaceuticals** (Grant/Research Support)**Roche TCRC** (Grant/Research Support)**Sagimet** (Grant/Research Support)**scPharmaceuticals Inc.** (Grant/Research Support)**Scynexis** (Grant/Research Support)**Spero Therapeutics** (Grant/Research Support)**TauRx Therapeutics** (Grant/Research Support)**Tetraphase Pharmaceuticals** (Grant/Research Support)**Theravance Biopharma Pharmaceutica** (Grant/Research Support)**USCAST** (Grant/Research Support)**VenatoRx** (Grant/Research Support)**Vical Incorporated** (Grant/Research Support)**Wockhardt Bio AG** (Grant/Research Support)**Zavante Therapeutics** (Grant/Research Support)**Zogenix International** (Grant/Research Support) **Haley Conde, B.S.**, **3-V Biosciences** (Grant/Research Support)**Achogen** (Grant/Research Support)**Amplyx Pharmaceuticals, Inc.** (Grant/Research Support)**Arixa Pharmaceuticals** (Grant/Research Support)**Arsanis Inc.** (Grant/Research Support)**B. Braun Medical Inc.** (Grant/Research Support)**Basilea Pharmaceutica** (Grant/Research Support)**BLC USA** (Grant/Research Support)**Boston Pharmaceuticals** (Grant/Research Support)**Bravos Biosciences, LLC** (Grant/Research Support)**Cidara Therapeutics Inc.** (Grant/Research Support)**Cipla, USA** (Grant/Research Support)**Corcept Therapeutics** (Grant/Research Support)**Cumberland Pharmaceuticals** (Grant/Research Support)**Debiopharm International SA** (Grant/Research Support)**Discuva Limited** (Grant/Research Support)**Emerald Lake Technologies** (Grant/Research Support)**Enhanced Pharmacodynamics** (Grant/Research Support)**Entasis Therapeutics** (Grant/Research Support)**E-Scape Bio** (Grant/Research Support)**Genentech** (Grant/Research Support)**Geom Therapeutics, Inc.** (Grant/Research Support)**GlaxoSmithKline** (Grant/Research Support)**Hoffmann-La Roche** (Grant/Research Support)**Horizon Orphan LLC** (Grant/Research Support)**ICPD Biosciences, LLC** (Grant/Research Support)**Indalo Therapeutics** (Grant/Research Support)**Insmed Inc.** (Grant/Research Support)**Institute for Clinical Pharmacodynamics** (Employee)**Iterum** (Grant/Research Support)**KBP Biosciences USA** (Grant/Research Support)**Kyoto Biopharma, Inc.** (Grant/Research Support)**Matinas** (Grant/Research Support)**Meiji Seika Pharma Co., Ltd.** (Grant/Research Support)**Melinta Therapeutics** (Grant/Research Support)**Menarini Ricerche S.p.A.** (Grant/Research Support)**Merck & Co., Inc** (Grant/Research Support)**Mutabilis** (Grant/Research Support)**Nabriva Therapeutics AG** (Grant/Research Support)**Naeja-RGM Pharmaceuticals** (Grant/Research Support)**Nosopharm SAS** (Grant/Research Support)**Novartis Pharmaceuticals Corp.** (Grant/Research Support)**NuCana Biomed** (Grant/Research Support)**Paratek Pharmaceuticals, Inc.** (Grant/Research Support)**Polyphor, Ltd.** (Grant/Research Support)**Prothena Corporation** (Grant/Research Support)**PTC Therapeutics** (Grant/Research Support)**Rempex Pharmaceuticals** (Grant/Research Support)**Roche TCRC** (Grant/Research Support)**Sagimet** (Grant/Research Support)**scPharmaceuticals Inc.** (Grant/Research Support)**Scynexis** (Grant/Research Support)**Spero Therapeutics** (Grant/Research Support)**TauRx Therapeutics** (Grant/Research Support)**Tetraphase Pharmaceuticals** (Grant/Research Support)**Theravance Biopharma Pharmaceutica** (Grant/Research Support)**USCAST** (Grant/Research Support)**VenatoRx** (Grant/Research Support)**Vical Incorporated** (Grant/Research Support)**Wockhardt Bio AG** (Grant/Research Support)**Zavante Therapeutics** (Grant/Research Support)**Zogenix International** (Grant/Research Support) **Sean Jones, B.S.**, **3-V Biosciences** (Grant/Research Support)**Achogen** (Grant/Research Support)**Amplyx Pharmaceuticals, Inc.** (Grant/Research Support)**Arixa Pharmaceuticals** (Grant/Research Support)**Arsanis Inc.** (Grant/Research Support)**B. Braun Medical Inc.** (Grant/Research Support)**Basilea Pharmaceutica** (Grant/Research Support)**BLC USA** (Grant/Research Support)**Boston Pharmaceuticals** (Grant/Research Support)**Bravos Biosciences, LLC** (Grant/Research Support)**Cidara Therapeutics Inc.** (Grant/Research Support)**Cipla, USA** (Grant/Research Support)**Corcept Therapeutics** (Grant/Research Support)**Cumberland Pharmaceuticals** (Grant/Research Support)**Debiopharm International SA** (Grant/Research Support)**Discuva Limited** (Grant/Research Support)**Emerald Lake Technologies** (Grant/Research Support)**Enhanced Pharmacodynamics** (Grant/Research Support)**Entasis Therapeutics** (Grant/Research Support)**E-Scape Bio** (Grant/Research Support)**Genentech** (Grant/Research Support)**Geom Therapeutics, Inc.** (Grant/Research Support)**GlaxoSmithKline** (Grant/Research Support)**Hoffmann-La Roche** (Grant/Research Support)**Horizon Orphan LLC** (Grant/Research Support)**ICPD Biosciences, LLC** (Grant/Research Support)**Indalo Therapeutics** (Grant/Research Support)**Insmed Inc.** (Grant/Research Support)**Institute for Clinical Pharmacodynamics** (Employee)**Iterum** (Grant/Research Support)**KBP Biosciences USA** (Grant/Research Support)**Kyoto Biopharma, Inc.** (Grant/Research Support)**Matinas** (Grant/Research Support)**Meiji Seika Pharma Co., Ltd.** (Grant/Research Support)**Melinta Therapeutics** (Grant/Research Support)**Menarini Ricerche S.p.A.** (Grant/Research Support)**Merck & Co., Inc** (Grant/Research Support)**Mutabilis** (Grant/Research Support)**Nabriva Therapeutics AG** (Grant/Research Support)**Naeja-RGM Pharmaceuticals** (Grant/Research Support)**Nosopharm SAS** (Grant/Research Support)**Novartis Pharmaceuticals Corp.** (Grant/Research Support)**NuCana Biomed** (Grant/Research Support)**Paratek Pharmaceuticals, Inc.** (Grant/Research Support)**Polyphor, Ltd.** (Grant/Research Support)**Prothena Corporation** (Grant/Research Support)**PTC Therapeutics** (Grant/Research Support)**Rempex Pharmaceuticals** (Grant/Research Support)**Roche TCRC** (Grant/Research Support)**Sagimet** (Grant/Research Support)**scPharmaceuticals Inc.** (Grant/Research Support)**Scynexis** (Grant/Research Support)**Spero Therapeutics** (Grant/Research Support)**TauRx Therapeutics** (Grant/Research Support)**Tetraphase Pharmaceuticals** (Grant/Research Support)**Theravance Biopharma Pharmaceutica** (Grant/Research Support)**USCAST** (Grant/Research Support)**VenatoRx** (Grant/Research Support)**Vical Incorporated** (Grant/Research Support)**Wockhardt Bio AG** (Grant/Research Support)**Zavante Therapeutics** (Grant/Research Support)**Zogenix International** (Grant/Research Support) **Ian A. Critchley, Ph.D.**, **Spero Therapeutics** (Employee, Shareholder) **Thomas R. Parr, Ph.D.**, **Spero Therapeutics** (Employee, Shareholder) **Nicole Cotroneo**, **Spero Therapeutics** (Employee, Shareholder) **Paul G. Ambrose, Pharm.D., FIDSA**, **3-V Biosciences** (Grant/Research Support)**Achogen** (Grant/Research Support)**Amplyx Pharmaceuticals, Inc.** (Grant/Research Support)**Arixa Pharmaceuticals** (Grant/Research Support)**Arsanis Inc.** (Grant/Research Support)**B. Braun Medical Inc.** (Grant/Research Support)**Basilea Pharmaceutica** (Grant/Research Support)**BLC USA** (Grant/Research Support)**Boston Pharmaceuticals** (Grant/Research Support)**Bravos Biosciences, LLC** (Grant/Research Support, Other Financial or Material Support, member/owner)**Cidara Therapeutics Inc.** (Grant/Research Support)**Cipla, USA** (Grant/Research Support)**Corcept Therapeutics** (Grant/Research Support)**Cumberland Pharmaceuticals** (Grant/Research Support)**Debiopharm International SA** (Grant/Research Support)**Discuva Limited** (Research Grant or Support)**Emerald Lake Technologies** (Grant/Research Support)**Enhanced Pharmacodynamics** (Grant/Research Support)**Entasis Therapeutics** (Grant/Research Support)**E-Scape Bio** (Grant/Research Support)**Genentech** (Grant/Research Support)**Geom Therapeutics, Inc.** (Grant/Research Support)**GlaxoSmithKline** (Grant/Research Support)**Hoffmann-La Roche** (Grant/Research Support)**Horizon Orphan LLC** (Grant/Research Support)**ICPD Biosciences, LLC** (Grant/Research Support, Other Financial or Material Support, member/owner)**Indalo Therapeutics** (Grant/Research Support)**Insmed Inc.** (Grant/Research Support)**Institute for Clinical Pharmacodynamics** (Employee)**Iterum** (Grant/Research Support)**KBP Biosciences USA** (Grant/Research Support)**Kyoto Biopharma, Inc.** (Grant/Research Support)**Matinas** (Grant/Research Support)**Meiji Seika Pharma Co., Ltd.** (Grant/Research Support)**Melinta Therapeutics** (Grant/Research Support)**Menarini Ricerche S.p.A.** (Grant/Research Support)**Merck & Co., Inc** (Grant/Research Support)**Mutabilis** (Grant/Research Support)**Nabriva Therapeutics AG** (Grant/Research Support)**Naeja-RGM Pharmaceuticals** (Grant/Research Support)**Nosopharm SAS** (Grant/Research Support)**Novartis Pharmaceuticals Corp.** (Grant/Research Support)**NuCana Biomed** (Grant/Research Support)**Paratek Pharmaceuticals, Inc.** (Grant/Research Support)**Polyphor, Ltd.** (Grant/Research Support)**Prothena Corporation** (Grant/Research Support)**PTC Therapeutics** (Grant/Research Support)**Rempex Pharmaceuticals** (Grant/Research Support)**Roche TCRC** (Grant/Research Support)**Sagimet** (Grant/Research Support)**scPharmaceuticals Inc.** (Grant/Research Support)**Scynexis** (Grant/Research Support)**Spero Therapeutics** (Grant/Research Support)**TauRx Therapeutics** (Grant/Research Support)**Tetraphase Pharmaceuticals** (Grant/Research Support)**Theravance Biopharma Pharmaceutica** (Grant/Research Support)**USCAST** (Grant/Research Support)**VenatoRx** (Grant/Research Support)**Vical Incorporated** (Grant/Research Support)**Wockhardt Bio AG** (Grant/Research Support)**Zavante Therapeutics** (Grant/Research Support)**Zogenix International** (Grant/Research Support)

